# American Medical Association

**Published:** 1875-06-15

**Authors:** Wm. B. Atkinson

**Affiliations:** Perm’t Sec’y, 1,400 Pine Street, Phila.


AMERICAN MEDICAL ASSOCIATION.—By request of many members, the Secretary will shortly issue a pamphlet edition of the MINUTES of the late session. All who desire copies, will please remit *fifty cents* at once, to WM. B. ATKINSON,
*Perm't Sec'y*,
1,400 Pine Street, Phila.

---

HEALTH OF DENVER.—Dr. F. G. Bancroft, city physician of Denver, Colorado, claims, in his annual report, the exceptionally favorable death-rate of 13 in 1,000 for that city. Of consumption he says :

I must say of the large number of consumptives who come to Colorado in all of the different varieties and stages of the disease, with the hope of an immediate and permanent cure, that the greater portion eventually die; yet I am fully of the opinion that the climate of Colorado gives to the invalid in the early stages better chances of either a complete restoration to health or a prolonged and happy life, than any other portion of the Union. Even in hopeless cases, our clear, dry, exhilarating air gives buoyancy to the spirits, that carries the patient to his final hour with less physical and mental suffering than could occur in damp and gloomy climates.—*Phil. Med.Rep.*

